# African polyvalent antivenom can maintain pharmacological stability and ability to neutralise murine venom lethality for decades post-expiry: evidence for increasing antivenom shelf life to aid in alleviating chronic shortages

**DOI:** 10.1136/bmjgh-2023-014813

**Published:** 2024-03-13

**Authors:** Gabriela Solano, Sinead Cunningham, Rebecca J Edge, Gina Duran, Adriana Sanchez, Mauren Villalta, Rachel H Clare, Mark C Wilkinson, Amy E Marriott, Camille Abada, Stefanie K Menzies, Molly Keen, David G Lalloo, Ymkje Stienstra, Michael Abouyannis, Nicholas R Casewell, Guillermo León, Stuart Ainsworth

**Affiliations:** 1 Instituto Clodomiro Picado, Universidad de Costa Rica, San Jose, Costa Rica; 2 Liverpool School of Tropical Medicine, Liverpool, UK; 3 Department of Infection Biology and Microbiomes, University of Liverpool, Liverpool, UK; 4 Malawi Liverpool Wellcome Trust Clinical Research Programme, Blantyre, Malawi; 5 Department of Internal Medicine/Infectious Diseases, University of Groningen, Groningen, The Netherlands

**Keywords:** Snake bite, stings and other envenoming

## Abstract

**Introduction:**

Antivenom is a lifesaving medicine for treating snakebite envenoming, yet there has been a crisis in antivenom supply for many decades. Despite this, substantial quantities of antivenom stocks expire before use. This study has investigated whether expired antivenoms retain preclinical quality and efficacy, with the rationale that they could be used in emergency situations when in-date antivenom is unavailable.

**Methods:**

Using WHO guidelines and industry test requirements, we examined the in vitro stability and murine in vivo efficacy of eight batches of the sub-Saharan African antivenom, South African Institute for Medical Research polyvalent, that had expired at various times over a period of 30 years.

**Results:**

We demonstrate modest declines in immunochemical stability, with antivenoms older than 25 years having high levels of turbidity. In vitro preclinical analysis demonstrated all expired antivenoms retained immunological recognition of venom antigens and the ability to inhibit key toxin families. All expired antivenoms retained comparable in vivo preclinical efficacy in preventing the lethal effects of envenoming in mice versus three regionally and medically important venoms.

**Conclusions:**

This study provides strong rationale for stakeholders, including manufacturers, regulators and health authorities, to explore the use of expired antivenom more broadly, to aid in alleviating critical shortages in antivenom supply in the short term and the extension of antivenom shelf life in the longer term.

**WHAT IS ALREADY KNOWN ON THIS TOPIC**

- The majority of antivenoms have shelf lives of 2–5 years post-manufacture, and once expired are typically discarded, despite antivenoms possibly retaining efficacy for many years post-expiry. Before this study, there was highly limited information on the retained efficacy of antivenoms for sub-Saharan Africa, the region which experiences the most acute shortages.

**WHAT THIS STUDY ADDS**

- Our study provides comprehensive, industry standard immunochemical and preclinical examination of eight expired batches of an antivenom, South African Institute for Medical Research polyvalent, that had expired at different times over the last 30 years. Our research demonstrates that antivenoms can retain key physiological and venom neutralising properties for up to 25 years, providing much needed evidence for the potential of expired antivenoms to be used in an emergency.

**HOW THIS STUDY MIGHT AFFECT RESEARCH, PRACTICE OR POLICY**

- We believe the evidence here, along with similar research performed by others in various key global regions, provides rationale for international stakeholders to explore whether current expiry dates of antivenom are too conservative, with the implication of potentially increasing antivenom shelf life to aid in alleviating chronic global antivenom shortages.

## Introduction

Snakebite envenoming, a WHO-recognised neglected tropical disease,^1^ is thought to cause approximately 81 000–138 000 deaths each year while permanently disabling a further 400 000 people.^2^ The victims of snakebite envenoming are overwhelmingly the poorest populations living in some of the most resource poor regions of the world.^3^ As it stands, the only established therapy for snakebite envenoming is antivenom, a polyclonal mixture of antitoxin antibodies refined from the sera of venom of hyperimmunised horses or sheep.

Antivenoms, like all therapeutics, have a defined shelf life. The WHO definition of shelf life is ‘the period of time, from the date of manufacture, that a product is expected to remain within its approved product specification while handled and stored under defined conditions’.^4^ For antivenoms, which are biologics, this period typically ranges from 2 to 5 years depending on the specific product, and is validated by testing the retention of key pharmacological, physicochemical, immunochemical and microbiological properties during the specified storage period. Once an antivenom has expired, it is outside the window of stability testing undertaken by the manufacturer, and thus the safety and effectiveness of the product become uncertain.

There have been critical shortages in antivenom supply for several decades.^5–7^ The shortages are a global phenomenon, but it is the regions in greatest need and most under-resourced that suffer the most acute shortages.^6 8^ Sub-Saharan Africa is the most notable example of this, with several antivenom market failures and frequent product stockouts, leading to extremely limited to no antivenom provision in large swathes of the continent.^8–10^ However, despite the shortfall in overall doses available, substantial quantities (up to 50% in one reported locale) of antivenom actually exceed their expiry dates and are subsequently discarded.^11–13^ The cause of this waste of critically precious antivenom is mainly due to difficulties in the national and regional antivenom inventory management, due to a lack of research and information around national requirements, weak infrastructure and financial inaccessibility, issues which are being actively addressed by various stakeholders.^14–16^

Considering the global crisis in antivenom supply, the loss of antivenom due to expiry has driven investigation into whether expired antivenoms retain clinical efficacy, with the rationale that they could be used in emergency situations when in-date antivenom is unavailable. It is widely recognised in academic settings that antivenoms can retain their efficacy for many years after their expiration, with several recent studies demonstrating retained antivenom preclinical efficacy, similar to that of non-expired antivenom, up to two decades post-expiry.^17–20^ Furthermore, there is clinical precedent for the use of expired antivenom in certain situations. For example, the US Food and Drug Administration (FDA) approved the use of expired North American coral snake antivenom after it was discontinued by its manufacturer Wyeth in 2006.^21^ In emergency cases, the use of ‘recently’ expired antivenom is recommended by the WHO if no other option is available.^22^ This advice seems to be reflected in practice, with reported cases of expired antivenoms being used in several countries, perhaps routinely, when in-date antivenoms were not available, with reported positive outcomes.^23 24^ The most extensive report detailing the use of expired antivenom was a clinical study of 31 patients suffering from systemic effects of envenoming in the Lao People’s Democratic Republic.^25^ The patients in this study received antivenom which was beyond expiry by 1–6 years, as a result of the unavailability of in-date antivenom within the country at the time.^25^

In the studies to date, there has been limited evaluation of the neutralising capacity of expired antivenom products for sub-Saharan Africa, arguably the region with the greatest antivenom supply crisis, with just a single study identified.^19^ South African Institute for Medical Research (SAIMR) polyvalent, manufactured by South African Vaccine Producers (SAVP), is currently the only polyvalent antivenom produced within sub-Saharan Africa^15^ and throughout its 50-year history, it has enjoyed a good reputation of clinical efficacy, despite limited published evidence to support this.^26^ Here, we present a preclinical analysis of the venom neutralising characteristics of expired SAIMR polyvalent antivenom, using eight expired batches going back to 1991 and using several of the most medically important venoms from the region. We also report on immunochemical analyses of product stability of these batches, using industry standard quality control assessments. Our findings demonstrate that SAIMR polyvalent can retain preclinical efficacy and maintain acceptable product stability up to 25 years after its stated expiry date.

## Methods

Additional detailed descriptions of all of the materials and methods used in this study are provided in the online supplemental methods.

10.1136/bmjgh-2023-014813.supp4 Supplementary data

### Antivenoms

For this study, we used the SAVP equine F(ab’)_2_ polyvalent antivenom, ‘SAIMR polyvalent antivenom’. SAIMR polyvalent is manufactured from horses that have been hyperimmunised with the venoms of *Bitis arietans*, *B. gabonica*, *Hemachatus haemachatus*, *Dendroaspis angusticeps*, *D. jamesoni*, *D. polylepis*, *Naja nivea*, *N. melanaluca* and *N. mossambica*. According to the product insert, SAIMR polyvalent antivenom is indicated as ‘effective against the venoms of the rinkhals, mambas, and all the cobras and vipers likely to cause life-threatening envenoming in Africa’. All SAIMR polyvalent antivenom used in this study was donated by the UK Health Security Agency (or its predecessors) shortly after its expiry date. Antivenoms were stored long term, unopened in their original sealed glass ampules, at 4°C. Eight batches of antivenom were used with the following expiry dates (month/year) and lot numbers: 08/1991 (lot A706 S1), 08/1994 (lot D04446), 07/1997 (lot G03146), 11/2000 (lot J06646), 09/2001 (lot K04846), 09/2012 (lot X02646), 07/2015 (lot BB01446), 11/2017 (lot BF00546). Transport of two vials of each batch of antivenom from Liverpool School of Tropical Medicine (LSTM) to Instituto Clodomiro Picado (ICP) was through a specialist courier service (Biocair) with uninterrupted refrigerated conditions (2–8°C) throughout. All vials were used immediately after opening. Prior to being opened, each ampoule was inspected for visual differences in its content. This included noting the colour and if there were any visible particulates of precipitates in the liquid. Due to limited quantity of antivenom of each batch available for analysis, all results below are representative of technical replicates (from single vials) only.

### Venoms

LSTM venoms: venoms used in ELISAs and in vitro snake venom metalloproteinase (SVMP) and phospholipase A_2_ (PLA_2_) assays were obtained from wild-caught specimens maintained in, or historical venom samples stored in, the Herpetarium of the LSTM. Following collection, venoms were immediately frozen and lyophilised to be stored as a powder at 4°C. Venoms were reconstituted in phosphate-buffered saline (25 mM sodium phosphate, 0.15 M sodium chloride (NaCl), pH 7.4) and stored at a concentration of 1 mg/mL at −20°C, unless stated otherwise. Venoms were only freeze-thawed once prior to use. Venoms used were pooled from multiple extractions of the following species: *B. arietans* (origin: Kenya), *D. polylepis* (Tanzania), *N. haje* (Uganda), *N. nigricollis* (Tanzania) and *H. haemachatus* (South Africa).

ICP venoms: venoms of adult specimens of *B. arietans* (unspecified origin, batch #322.061), *D. polylepis* (unspecified origin, batch #416.031) and *N. nigricollis* (unspecified origin, batch #616.031) were purchased from Latoxan (Portes-dès Valence, France). After collection, venoms were stabilised by lyophilisation and stored at −40°C. Solutions of venoms were prepared in 0.9% NaCl injection USP, immediately before use.

### Nephelometric turbidity

Turbidity of antivenoms was measured at ICP in 2023. Nephelometric turbidity of antivenoms, expressed as nephelometric turbidity units (NTUs), was assessed as per US Pharmacopeia specifications.^27^ Briefly, samples of antivenoms were placed in reading cells and analysed in a LaMotte 2020 Turbidimeter (Chestertown, Maryland, USA) through comparing the intensity of light scattered by the sample with the intensity of the light scattered by a reference solution. Assays were performed in triplicate and results expressed as mean±SD.

### SVMP assay

The SVMP assay was performed at LSTM in 2022 as previously described.^28 29^ Briefly, 1 µg of venom in a 1 µL volume was added to each well, followed by 10 µL of antivenom diluted in assay buffer (150 mM NaCl, 50 mM Tris pH 7.5). Antivenom dilutions of 1 in 4, 1 in 8 and 1 in 16 were used. The negative control was assay buffer alone. Each condition was performed in quadruplicate on a 384-well plate (Greiner Bio-One). The plates were briefly spun at 2500 rpm and then incubated for 25 min at 37°C, and for a further 5 min at room temperature. 90 μL of a quenched fluorogenic substrate (ES010, R&D Biosystems, supplied as a 6.2 mM stock) was then added to each well using a Multidrop Combi (Thermo-Scientific), and reactions monitored using a CLARIOstar Plus plate reader (BMG Labtech) at 420 nm for 75 min (10 flashes/well, 100 cycles) at an excitation wavelength of 320 nm and emission wavelength of 405 nm. The measurements at 52 min were chosen as the endpoint as all fluorescence curves had plateaued by this time point. Raw data were recorded in MARS data analysis software (BMG Labtech) prior to export and analysis in GraphPad Prism V.9. Results were then expressed as a percentage of the venom-only SVMP activity.

### PLA_2_ assay

The PLA_2_ assay was performed at LSTM in 2022. The antivenoms were tested using the commercial Abcam sPLA_2_ assay (Abcam) optimised for high-throughput screening.^30^ The final reaction consisted of 9 µL each of the antivenoms (n=8) against 1 µL of each snake venom plated out into a 384-well plate (Grenier Bio). For *H. haemachatus* and *N. nigricollis,* stock solutions of 10 mg/mL were diluted 1 in 2000, resulting in 5 ng of venom per reaction. Following incubation of the venom and antivenoms for 30 min at 37°C protected from light, the plate was acclimatised for 5 min to room temperature. 5 µL of a stock of 4 mM 5,5′-Dithiobis(2-nitrobenzoic acid) (DNTB) in distilled water was added to each well of the venom+antivenom plate. The substrate 1 mM stock was prepared by resuspending in 1X assay buffer (diluted from the 10X stock in MilliQ water: 25 mM Tris-hydrogen chloride, pH 7.5, 10 mM calcium chloride, 100 mM potassium chloride, 0.3 mM Triton X-100). The addition of 30 µL of the substrate solution per well resulted in a final reaction concentration of 0.89 mM. The addition of venom, DTNB and substrate was done using a VIAFLO384 liquid handler (Integra). Following the addition of the substrate, the plates were immediately read kinetically on a CLARIOstar plate reader at 405 nm for 15 min (settings for a full 384-well plate were 11 flashes, 161 s cycle time). Raw data were recorded in MARS data analysis software prior to export and analysis in GraphPad Prism V.9. Results were then expressed as a percentage of the venom-only PLA_2_ activity.

### In vivo neutralisation of venom-induced lethality

#### Sample size determination

We used the WHO-recommended essential in vivo preclinical assay to evaluate the ability of expired antivenoms to neutralise venom-induced lethality.^31^ This assay stipulates minimum numbers of animals required per assay to gain statistical significance and normal data distribution via Probit analysis of the median effective dose (ED_50_).^32^ This requires data from five experimental groups (consisting of five experimental units, that is, five mice), which receive a fixed dose of venom with a variable dose of antivenom. The groups are required to have one group with all survivors, one with all deaths and three groups with some survivors and some deaths. Therefore, five experimental groups are required for each ED_50_ assay. To determine the in vivo efficacy of each of the eight antivenoms versus three medically important venoms requires 24 assays (600 mice).

#### Animal maintenance

CD1 mice weighing 20–22 g, of both sexes, were obtained from the Bioterium of ICP. Mice were housed by sex in randomly allocated groups of five in Techniplast Eurostandard Type II 1264C cages (L25.0×W40.0×H14.0 cm) and maintained at 18–24°C, 60–65% relative humidity and 12:12 light-dark cycle, with food and water available ad libitum.

#### Neutralisation of lethality assessment

All experiments used mixed genders, and experimenters were unblinded to the test articles. Mice were pretreated with the analgesic tramadol, administered subcutaneously, at a dose of 50 mg/kg,^33^ 15 min prior to administration of venom challenge. Mice were then injected intravenously with mixtures containing a challenge dose of venom dissolved in sterile 0.9% NaCl USP injection solution and variable dilutions of antivenom, which were premixed and incubated at 37°C for 30 min (volume of injection was 0.2 mL).^34^ Challenge doses were three times the previously determined median lethal dose (LD_50_) for the venoms of *N. nigricollis* (3× LD_50_=55.2 µg/mouse)^35^ and *D. polylepis* (3× LD_50_=1.14 µg/mouse)^36^ and five times for the venom of *B. arietans* (5× LD_50_=110 µg/mouse).^37^ The rationale for using 3× LD_50_ challenge doses for *N. nigricollis* and *D. polylepis*, rather than the conventional 5× LD_50_, was a refinement due to experience with these particular venoms demonstrating the use of 5× LD_50_ resulted in poor resolution of dose groups outcomes. The number of resulting deaths was recorded at 6 hours.^38^ The ED_50_ and the corresponding 95% CIs were calculated by Probit analysis.^32^ In line with recent WHO recommendations to report in vivo antivenom efficacy outcomes using the potency metric^39^ (the amount of venom completely neutralised per millilitre of antivenom (mg/mL), resulting in 100% survival of test animals^40^), calculated ED_50_ values were used to determine potency using the following equation: p=n–1 LD_50_/ED_50_, where n=the number of LD_50_ in the challenge dose.

## Results

### Total protein concentration

**Figure 1:**
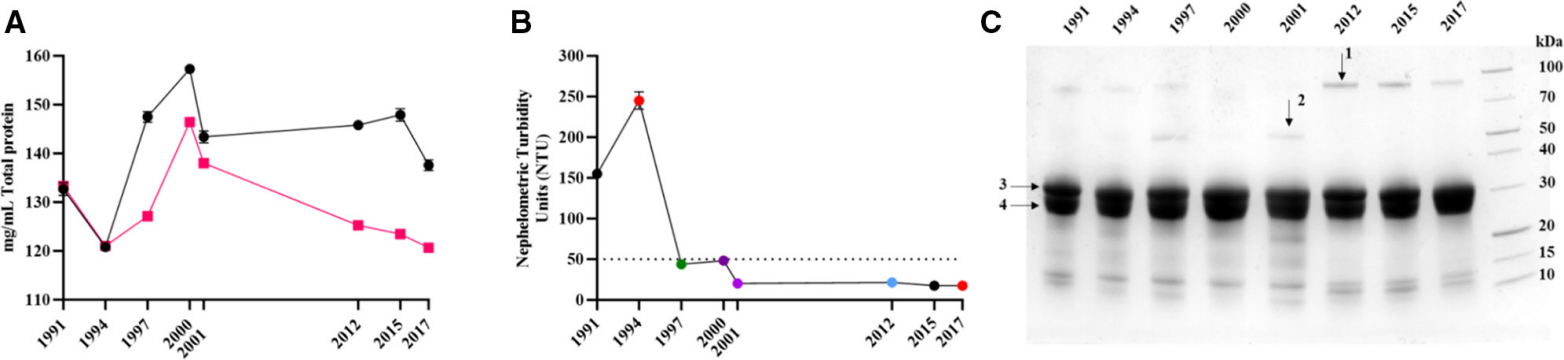
Physiochemical analysis of expired SAIMR polyvalent antivenoms. Antivenom expiry year is represented on each x axis. (A) Mean (n=3 technical replicates) total protein concentration (mg/mL) of each batch determined by Biuret (black points) or Nanodrop (red points). Error bars represent the ±SD. (B) Nephelometric turbidity assessment of each batch of antivenom expressed as mean (n=3) nephelometric turbidity units (NTUs). Error bars represent the ±SD. The dotted line represents 50 NTUs, the maximum permitted NTU for antivenom manufactured at Instituto Clodomiro Picado. (C) Reducing SDS-PAGE profiles of expired SAIMR polyvalent antivenom batches (denoted by year of expiry along top) centrifuged and the resulting supernatants diluted 1/75 prior to loading. Arrows represent bands analysed by mass spectrometry. 1=alpha-1-antitrypsin, 2=alpha-2-macroglobulin, 3 and 4=immunoglobulin fragments representative of antibody heavy and light chains, respectively. SAIMR, South African Institute for Medical Research.

Total protein concentration of the eight antivenoms was determined by Biuret assay and ultraviolet/visible (UV/VIS) spectrometry. Concentrations measured by the two methods were consistent overall, although the OD_280_ nm measurements typically reported lower concentrations than Biuret, with no trend in protein concentration over time apparent (figure 1A). Total protein concentration ranged from a low of 121 mg/mL (1994) to 157 mg/mL (2000) by Biuret (mean=142 mg/mL, SD=11 mg/mL) or 121 mg/mL (1994) to 146 mg/mL (2000) by UV/VIS spectrometry (mean=129 mg/mL, SD=9 mg/mL).

### Turbidity

Visual inspection of the eight unopened antivenom vials revealed the oldest vial (1991) appeared cloudy with fine particulate matter, while the 1994 expiry antivenom appeared to have larger particulate matter but were transparent and pale yellow in colour. The remaining six antivenoms were transparent and pale yellow in colour. Nephelometric turbidity analysis reflected this, with elevated values for the oldest vials from 1991 and 1994, but acceptable levels of turbidity (<50 NTUs) for the remaining eight vials despite the time lapsed after their expiry (figure 1B).

### Protein profiles

SDS-PAGE profiles of all antivenoms and GFC of four antivenoms demonstrated that >90% of the antivenom protein content corresponds to F(ab’)_2_ immunoglobulin fragments (ie, the active ingredients) (figure 1C). Overall, results did not demonstrate any substantive evidence of immunochemical degradation over time. Additional bands and peaks not corresponding to F(ab’)_2_ fragments were visible in both SDS-PAGE and GFC. Mass spectrometry identification of these additional bands reveals they are the common serum proteins alpha-1-antitrypsin and albumin. GFC also revealed an additional peak at 214 nm but not at 280 nm, indicating that it is not proteinaceous in nature (online supplemental figure 1).

10.1136/bmjgh-2023-014813.supp1 Supplementary data

### ELISA and immunoblotting

**Figure 2:**
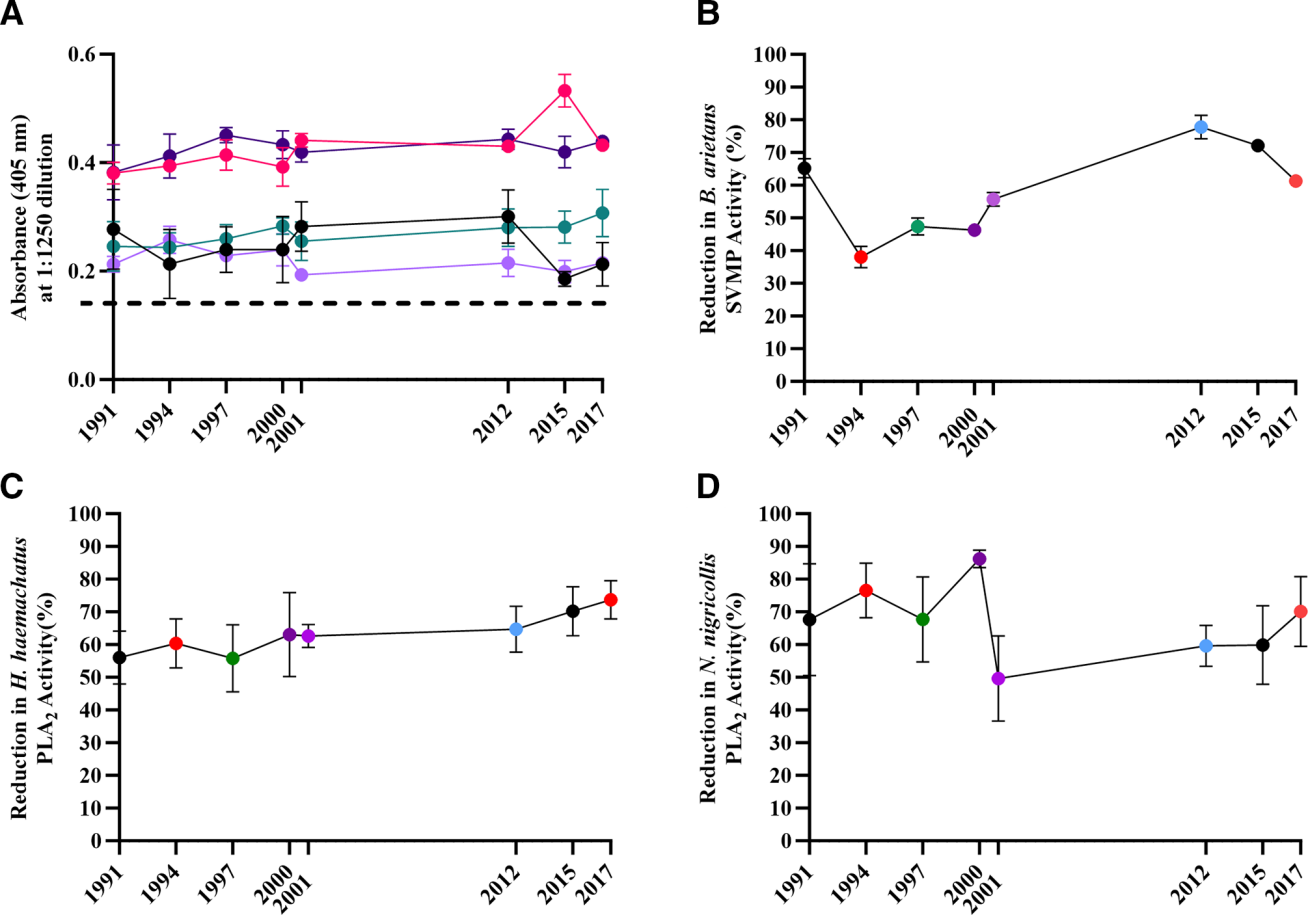
In vitro preclinical analysis of expired SAIMR polyvalent batches. Antivenom expiry year is represented on each x axis. (A) Mean (n=3 technical replicates) antibody titre at a neat antivenom dilution of 1:1250, expressed as absorbance at 405 nm. Error bars=±SD. Light purple =*Bitis arietans* venom, dark purple =*Dendroaspis polylepis,* red =*Hemachatus haemachatus*, green =*Naja haje*, black =*N. nigricollis,* dashed line=blank (PBS). (B) Ability of each antivenom to inhibit in vitro SVMP activity of *B. arietans* venom. Results represent mean (n=3 technical replicates) % inhibition compared with activity of venom-only controls. Error bars=±SD. (C,D) Ability of each antivenom to inhibit in vitro PLA_2_ activity of *H. haemachatus* (C) and *N. nigricollis* (D). Points represent mean (n=8 technical replicates) % inhibition compared with the activity of venom-only controls. Error bars=±SD. PBS, phosphate-buffered saline; PLA_2_, phospholipase A_2_; SAIMR, South African Institute for Medical Research; SVMP, snake venom metalloproteinase.

Next, we examined the ability of antivenoms to recognise, via ELISA, five key African venoms; *B. arietans, H. haemachatus, D. polylepis, N. haje and N. nigricollis*. All expired antivenoms were able to recognise the five venoms (figure 2 and online supplemental figure 2), with examination of the absorbance at OD_405_ nm at a dilution of 1 in 1250, representing a dilution mid-titration curve, proving to be consistent over time (figure 2A). Immunoblotting against the same venoms suggested that there was an overall decrease in recognition of venoms by all antivenoms which expired from 1991 to 2001, as compared with antivenoms which expired in 2012, 2015 and 2017 (online supplemental figure 3).

10.1136/bmjgh-2023-014813.supp2 Supplementary data

10.1136/bmjgh-2023-014813.supp3 Supplementary data

### Antivenom in vitro neutralising capacity

We subsequently investigated the ability of the expired antivenoms to neutralise toxin-specific activity in biochemical functional assays, specifically PLA_2_ activity (in *H. haemachatus* and *N. nigricollis* venoms) and SVMP activity (in *B. arietans* venom).

The SVMP activity of *B. arietans* venom was inhibited by all antivenoms to varying extent, with the 2012 expiry antivenom having the most potent SVMP-inhibiting activity (77.8%), while the 1994 batch possessed the lowest level of SVMP inhibition (38.0%) (figure 2B). There is a gradual reduction in *B. arietans* SVMP-inhibiting capability in ageing antivenoms from 2017 to 1994. However, the oldest antivenom (1991 expiry) has a greater inhibiting capability than the majority of antivenoms, with the exception of the 2012 and 2015 expiry.

The functional PLA_2_ activity of both *H. haemachatus* and *N. nigricollis* was inhibited by all antivenoms to varying extent (55.8–73.7% for *H. haemachatus* and 49.7–86.2% for *N. nigricollis*) (figure 2C,D). However, the capability of individual expired antivenoms in inhibiting either *H. haemachatus* or *N. nigricollis* PLA_2_ activity was not consistent across the two venoms. The PLA_2_-inhibiting capacity of the antivenoms versus *H. haemachatus* venom steadily reduced as the antivenoms aged (figure 2C), while antivenoms which expired in or before 2000 appeared substantially more capable of inhibiting *N. nigricollis* PLA_2_ activity than the more recently expired antivenoms (figure 2D).

### Antivenom in vivo neutralising capacity

**Figure 3:**
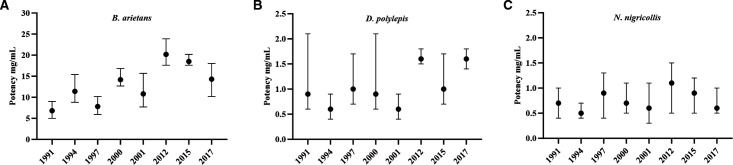
The ability of expired SAIMR polyvalent antivenoms to neutralise murine in vivo lethality. Antivenom expiry year is represented on each x axis. The ability to neutralise murine lethality is expressed as the median neutralising dose (ED_50_), presented as mg of venom neutralised per mL of antivenom (mg/mL) calculated by Probit analysis. Venoms tested were (A) *Bitis arietans* (vs 5× LD_50_), (B) *Dendroaspis polylepis* (vs 3× LD_50_) and (C) *Naja nigricollis* (vs 3× LD_50_). Error bars represent 95% Cls. For all experiments, each venom and antivenom dose were premixed, incubated at 37°C for 30 min before intravenous administration (n=5 mice/group). Results are representative of 6 hours post-venom injection. ED_50_, median effective dose; LD_50_, median lethal dose; SAIMR, South African Institute for Medical Research.

With the demonstration that expired antivenoms retained in vitro venom toxin neutralising capability, despite substantial time passing since expiry, we proceeded to determine the murine in vivo ED_50_ and subsequently the potency, of each expired antivenom. Using the WHO-recommended essential in vivo preclinical assay to measure antivenom neutralisation of venom-induced lethality,^31^ we assayed three medically important venoms (*B. arietans, D. polylepis* and *N. nigricollis*) whose envenoming is indicated for treatment by SAIMR polyvalent. Results demonstrate all antivenoms retained their ability to neutralise the systemic lethal effects of each venom (figure 3 and online supplemental table 1). The mean potency values for *B. arietans* were variable, with some batches having distinct lower (1991 and 1997) or higher (2012 and 2015) potency, with the remainder having potency broadly spread in between (figure 3A). Overall, while significant differences in potency were notable between some batches, there was no correlation with the *B. arietans* venom neutralising ability with age or protein content. The majority of mean potency values of the expired antivenoms versus *N. nigricollis* and *D. polylepis* venoms were substantially lower than those versus *B. arietans* venom, in line with the well-described weaker capability of antivenoms in neutralising elapid venoms versus that of viper venoms (figure 3).^41^ The majority of expired antivenoms had potency values in the range of 0.6–1 mg/mL with substantially overlapping CIs against *D. polylepis* venom, while 2012 and 2017 expiry had notably greater potency (1.6 mg/mL) (figure 3B). As with the expired antivenom potency versus *B. arietans* venom, while significant differences in potency were notable between individual batches, overall, there was no correlation with *D. polylepis* venom neutralising ability with either age of the expired antivenom or its protein content. Expired antivenom potency versus *N. nigricollis* venom demonstrated remarkable consistency over time, with mean potency in the range of 0.6–1.1 mg/mL with broadly overlapping CIs, with no correlation with time of antivenom expiry or protein content (figure 3C).

10.1136/bmjgh-2023-014813.supp5 Supplementary data

## Discussion

In this study, we examined the immunochemical and preclinical neutralising efficacy of eight batches of expired SAIMR polyvalent antivenom, ranging from 6 to 32 years post-expiry, which had been kept in long-term refrigerated conditions, with the objective of inferring their potential suitability for use when alternatives are unavailable and to explore if existing expiry dates could be extended. The key finding of this study is that SAIMR polyvalent can retain the in vivo ability to neutralise murine venom-induced lethality for over 30 years; however, physical stability is limited to a shorter period of up to 25 years.

A major consideration for clinicians using antivenom is its visual appearance, with most clinicians unlikely to administer cloudy antivenoms for fear of loss of activity or increased likelihood of adverse reaction.^42^ In terms of physical stability, only the oldest antivenoms, which expired in 1991 and 1994, were visibly cloudy, with levels of turbidity in excess of permissible quality control levels. The turbidity is assumed to be aggregation of F(ab’)_2_, which does not represent a loss in neutralising capacity in some of the tests presented here, likely due to the majority of F(ab’)_2_ present in antivenom not being clinically relevant for envenoming,^43^ but is enough to increase turbidity substantially. Antivenom total protein concentration broadly remained stable over time, and SDS-PAGE analysis demonstrated comparable levels of F(ab’)_2_ fragments (figure 1C). However, minor differences in protein profiles were notable, indicating various levels of impurities, which we consider to be a result of normal manufacturing variability as opposed to deterioration in product.

Prior to in vivo assays, all antivenom batches were examined using in vitro immunological and biochemical assays to assess their ability to recognise and neutralise venom toxins. ELISA and immunoblots demonstrated all expired batches were able to recognise venoms, with no substantial differences in recognition based on antivenom expiry. Biochemical in vitro assays demonstrated that all antivenoms retained the ability to partially neutralise SVMP and PLA_2_-specific activity. Apparent decreases in SVMP and PLA_2_ neutralising activity for *B. arietans* and *H. haemachatus* were noted, with older expiring antivenoms inhibiting less toxin activity compared with antivenoms expiring more recently, although this trend was not apparent for *N. nigricollis* PLA_2_ activity. It is important to note that while these assays are a valuable indicator of whether an antivenom may or may not be suitable for use in vivo, thus preventing the use of mice in experiments likely to fail, they are not capable of predicting actual performance in vivo.

The results of the ED_50_ assay for determining the potency of each antivenom’s ability to neutralise the lethal effects of *D. polylepis*, *N. nigricollis* and *B. arietans* venom demonstrated all expired antivenoms retained preclinical efficacy, with no evidence of degradation of capability over time since expiry. The ED_50_ and potency values of the antivenoms versus *D. polylepis* and *N. nigricollis* are highly similar to that of previously reported values for these venoms,^44–47^ whereas the values for *B. arietans* are all within the range (higher end) of values previously reported.^44 46–48^ Importantly, the potency values for *B. arietans* and *D. polylepis* are highly similar to that established for a non-expired batch of SAIMR polyvalent (Batch BL01646, expiry May 2023) tested in 2021 versus *B. arietans* and *D. polylepis* venom from eSwatini (p=20.3 mg/mL and 1.8 mg/mL, respectively),^44^ suggesting that the in vivo potency of the expired antivenom batches is comparable with that of non-expired batches.

As described, many studies have demonstrated retained efficacy of expired antivenoms,^17–19^ and it is important to consider whether existing antivenom stocks could be used more efficiently,^16^ particularly given the lack of access to these products in low and middle-income settings.^49–51^ Reports suggest that clinicians are already using expired antivenom products due to lack of availability of in-date products,^25^ and the South-East Asia regional office WHO guidelines for managing snakebite endorse this in certain circumstances: ‘in patients with severe envenoming, recently expired antivenoms may be used if there is no alternative’.^22^ However, we must emphasise that while we and others have demonstrated retained preclinical efficacy of expired antivenoms in mouse models, the clinical efficacy and safety of these antivenoms cannot be confirmed from these assays. Expired antivenoms which are cloudy or have precipitates must not be administered due to the likely high risk of adverse reactions resulting from aggregates.^52^ The legal implications of prescribing expired antivenom must be emphasised, as the prescribing clinician may subsequently become liable for any adverse events suffered by the patient following administration of an expired antivenom and would need to counsel the patient on the potential risks.

Currently available antivenoms, particularly those marketed in Asia and Africa, are of varying quality and have often been approved by regulators and provisioned by health services, despite a lack of preclinical or clinical efficacy data.^26 41 46 50^ This lack of regulation has been disastrous, with evidence suggesting that poor-quality products have caused harm to patients.^53 54^ Some of the methods used in the present study are similar to those applied by the WHO antivenom risk–benefit assessment, which endeavours to exclude poor-quality products from the market. This programme includes a review of the product dossier, laboratory analysis (according to WHO standards for biological products)^31^ and manufacturing site inspection (to ensure adherence to Good Manufacturing Practices). Thus, it may be feasible for the WHO antivenom risk–benefit assessment to be extended to evaluate the quality and preclinical efficacy of expired products, which could enable manufacturers of good-quality antivenoms to extend the expiry date, or could provide regulators and clinicians with assurance that certain products can be administered for a defined period after their expiry date has passed. Preferentially, and following strengthening of regulatory systems in low/middle-income country settings, organisations such as the proposed African Medicines Agency^55^ could lead on ensuring the quality of antivenom products is acceptable, and on defining appropriate expiry dates.

There would be particular value in extending antivenom expiry dates if the proposed WHO antivenom stockpile for Africa were to become established.^56^ Efforts in the USA to stockpile medications that protect against certain public health emergencies,^57^ namely pandemics, have been hindered by the high costs of regularly replacing expired stockpiled products, which are carefully stored, but not used, for long periods of time. In response to this challenge, the FDA has provided various routes for products to be approved for use beyond their initial expiry date.^58^ Given that a regional stockpile of antivenom would entail storage of a large volume of product in a controlled environment, with appropriate temperature regulation, it is feasible that products could remain safe and effective beyond their expiration date, which could improve the financial viability of this important project. Additional in vivo preclinical evaluation of antivenom products would incur a substantial financial cost but given that antivenom products are expensive to produce,^9^ this may be cost-effective. The ethical cost of increasing what is already substantial and severe, in vivo preclinical testing would also have to be carefully considered. Many manufacturers will have run real time and accelerated stability studies on their antivenom products to explore what duration of time is acceptable for expiry (ie, beyond the listed expiry). Going forward, manufacturers could consider making these data available, such as described by Morokuma *et al*,^20^ to provide additional confidence in extended expiration.

Potential wider disincentives for manufacturers to pursue extension of antivenom expiry dates must be considered. The antivenom market has been fragile and manufacturers of higher-quality products have previously ceased production due to unsustainable economics,^54^ and there is a theoretical risk that extension of expiry dates could reduce product turnover and further impair financial sustainability. Nevertheless, by reducing waste of antivenom products, it is likely that manufacturers could adjust prices to offset losses while ultimately reducing the cost to health services and the public. The benefit of evaluating products based on quality, which has been pioneered by the WHO antivenom risk–benefit assessment, and removing poor products from the market, is a major step forward and should be further expanded to define evidence-based expiry dates.

A major limitation in this study is the absence of any non-expired SAIMR polyvalent for direct comparison. The reasoning behind this is twofold. First, SAIMR polyvalent is extremely expensive and notably extremely difficult to procure outside of South Africa, with anecdotal evidence of worsening supply issues recently. Second, due to these supply issues, we considered it unethical to use the highly limited quantity of SAIMR polyvalent available to us for research purposes, when they may be required in the event of an envenoming. Despite this, comparison with recent in vivo preclinical data available examining the efficacy of non-expired SAIMR polyvalent versus two similar venoms used in this study, demonstrating highly similar results,^44^ provides confidence in our conclusion that the expired antivenoms in this study have no demonstrable reduction in preclinical efficacy due to age.

Clearly, the older antivenoms used in this study (1991 and 1994 expiry) would not be considered for clinical use based on their appearance alone. Our results clearly suggest that the apparent instability associated with storage time is not reflected as a change in immunochemical properties or the neutralising potency, but as an increment of the turbidity. Reflecting this, antivenoms from 1997 onwards have favourable quality control profiles largely indistinguishable from what would be expected for in-date products. Therefore, we believe this study provides strong rationale for stakeholders (manufacturers, regulators and health authorities) to explore the use of expired antivenom and the extension of antivenom shelf life. The use of expired antivenom needs to be carefully assessed on a product-by-product and venom-by-venom basis, and confirmation of safety of such products is necessary. However, the evidence, both preclinical^17–19^ and clinical,^21 23 25^ demonstrating the long-term retained efficacy of antivenoms should lead to initiatives regarding changing expiry dates that could assist in alleviating chronic antivenom availability shortages globally.

## Data Availability

Data are available upon reasonable request. All raw data are freely available upon request to the corresponding authors.
