# Supplementary material for: African polyvalent antivenom can maintain pharmacological stability and ability to neutralise murine venom lethality for decades post-expiry: evidence for increasing antivenom shelf life to aid in alleviating chronic shortages
Source: BMJ Glob Health. 2024 Mar 13;9(3):e014813. doi: 10.1136/bmjgh-2023-014813 (PMC10941113; doi:10.1136/bmjgh-2023-014813)
Supplement: Supplementary data [file bmjgh-2023-014813supp004.pdf]

SUPPLEMENTARY METHODS

Total protein determination

Total protein content of antivenoms were determined by two methods, colorimetric and spectrophometric, as suggested in the WHO Technical Report Series 1004; *Guidelines for the production, control and regulation of snake antivenom immunoglobulins.*[1]

Biuret method

Total protein concentration was determined by a Biuret method at ICP in 2023. The modified Biuret method was performed by adding 50 µL of BSA standard or each antivenom sample to 2500 µL of Biuret reagent (1200 mM NaOH, 32 mM $KNaC_4H_4O_6$, 12 mM $CuSO_4$ and 30 mM KI).[2] Samples were incubated for 10 minutes at 37 ˚C, and measured spectrophotometrically at 540 nm on a UV-1800 Shimadzu UV Spectrophotometer. Assays were performed in triplicate. Results were calculated from a calibration curve of the standards (Sigma-Aldrich, P5494) used and expressed as mean ± standard deviation (SD).

UV/VIS spectrometry

Total protein spectrometry analysis was performed at LSTM in 2022. Total protein concentration was also determined by A280 UV/VIS spectrometry using a NanoDrop One (Thermo Scientific) spectrophotometer. Briefly, the spectrophotometer was blanked using PBS (25 mM sodium phosphate, 0.15 M NaCl, pH 7.4), prior to application of 1 µL of undiluted antivenom which was analysed at A280 using the in-built IgG mass extinction coefficient (13.7 L/gm-cm). Readings were repeated in triplicate and expressed as mean ± SD.

SDS-PAGE

SDS-PAGE was performed at LSTM in 2023. Samples were prepared for reducing SDS-PAGE analysis by adding sample buffer to a final concentration of 2% SDS, 5% [illegible]-mercaptoethanol and then heating at 85 °C for 5 mins. Electrophoresis was performed on 4-20% acrylamide gels (BioRad) using a Tris-glycine buffer system (Laemmli, 1970), alongside a PageRuler Unstained Broad Range Protein Ladder (ThermoFisher) followed by staining with Coomassie Blue R250.

Enzyme-linked immunosorbent assay (ELISA)

ELISA was performed at LSTM in 2022. Venoms were diluted in 100 mM carbonate-bicarbonate coating buffer pH 9.6 and coated at 100 ng per well in 96-well ELISA plates (Nunc

Solano G, *et al. BMJ Glob Health* 2024; 9:e014813. doi: 10.1136/bmjgh-2023-014813

MaxiSorp, ThermoScientific) and incubated overnight at 4˚C. The following day, plates were washed three times with tris-buffered saline (TBS [50 mM Tris-Cl, 0.15 M NaCl, pH 7.4]) supplemented with 0.1% Tween-20 (TBS-T), before being blocked with 5% non-fat milk in TBS-T and incubated for 2 hours at room temperature. Plates were washed again three times with TBS-T followed by the addition of primary antibodies (neat antivenom and normal horse IgG [BioRad] as negative control) in duplicate, at an initial dilution of 1 in 100 in blocking buffer, which were five-fold serially diluted across the plate and incubated at 4˚C overnight. The following day plates were washed three times with TBS-T and incubated for 2 hours at room temperature with secondary antibody horseradish peroxidase-conjugated rabbit anti-horse IgG (Sigma A6917), diluted to 1 in 1,000 in PBS (25 mM sodium phosphate, 0.15 M NaCl, pH 7.4). Plates were washed three times with TBS-T followed by the addition of substrate (3% 2,2'-azino-bis(3-ethylbenzothiazoline-6-sulfonic acid), Sigma) in citrate buffer pH 4.0 containing 0.1% hydrogen peroxide. Plates were incubated at room temperature for 15 minutes to develop, and the signal was measured spectrophotometrically at 405 nm on an LT-4500 plate reader (Labtech).

Mass spectrometry

Mass spectrometry was performed at ICP in 2023. SDS-PAGE protein bands were excised from gels and in-gel digested overnight with sequencing-grade trypsin after reduction with 10 mM dithiothreitol and alkylation with 50 mM iodoacetamide, using an automated workstation (Intavis). The resulting peptides were dried, redissolved in water with 0.1% formic acid, and submitted to nano-LCMS/MS. Five µL of digest were loaded on a C18 trap column (75 µm × 2 cm, 3 µm particle; PepMap®, Thermo), washed with 0.1% formic acid (solution A), and separated at 200 nL/min with a 3 µm particle, 15 cm × 75 µm C18 Easy-spray® analytical column using a nano-Easy® 1200 chromatograph (Thermo). A gradient from 0.1% formic acid (solution A) to 80% acetonitrile with 0.1% formic acid (solution B) was developed: 1–5% B in 1 min, 5–25% B in 30 min, 25–79% B in 6 min, 79–99% B in 2 min, and 99% B in 6 min, for a total time of 45 min. MS spectra were acquired in positive mode at 1.9 kV, with a capillary temperature of 230 °C, using 1 µscan at 400–1600 m/z, maximum injection time of 100 msec, AGC target of 3 × 106 , and orbitrap resolution of 70,000. The top 10 ions with 2–4 positive charges were fragmented with AGC target of 1 × 105 , maximum injection time of 110 msec, resolution 17,500, loop count 10, isolation window of 1.4 m/z, and a dynamic exclusion time of 5 s[3] MS/MS spectra were processed for peptide matching with protein sequences contained in the UniProt/SwissProt database for Horse (June 2023), using Peaks X® (Bioinformatics

Solutions). Cysteine carbamidomethylation was set as a fixed modification, while deamidation of asparagine or glutamine and methionine oxidation were set as variable modifications, allowing up to 3 missed cleavages by trypsin. Parameters for match acceptance were set to FDR≤0.1%, -10 lgP protein score ≥100.

Gel filtration chromatography

Gel filtration chromatography (GFC) was performed at LSTM in 2022. For analysis of native molecular weight, a 24 mL Superdex 200HR column was set up on an AKTA LC system (Cytiva) and equilibrated in PBS (25 mM sodium phosphate, 0.15 M NaCl, pH 7.4). The column was operated at a flow rate of 0.5 mL/min and elution was monitored at 280 nm. Fifty µL of a 0.5 mg/mL antivenom sample was centrifuged at 14,000 x g for 10 mins and immediately injected onto the column. The column was calibrated by running 50 µL of BioRad SEC standard under the same condition and the molecular weights of the peaks observed in the antivenom samples were calculated from the calibration curve.

Immunoblotting

Immunoblotting was performed at LSTM in 2022. Venoms were diluted in PBS to 0.2 µg/µL and equal volume of 2 × denaturing buffer (100 mM Tris-Cl pH 6.8, 20% v/v glycerol, 4% SDS, 0.2% bromophenol blue, 100 mM dithiothreitol) was added. Samples were incubated at 100 °C for 5 minutes then loaded onto a MiniPROTEAN TGX 4–20% gel (BioRad) for protein separation, then transferred to nitrocellulose membrane (BioRad) using the TransBlot Turbo system mixed molecular weight programme. PageRuler Prestained protein ladder (ThermoFisher) was used as a molecular weight marker. Protein loading was visualised using Revert 700 Total Protein Stain (LI-COR Biosciences) according to manufacturer's instructions and imaged in the 700 nm channel for 2 min on an Odyssey Fc imaging system (LI-COR Biosciences). Membranes were blocked for 1 hour at room temperature on an orbital shaker in 5% rabbit serum in TBS-T. Membranes were incubated with antivenom overnight at 4 °C on an orbital shaker at 1 in 5000 dilution in blocking solution. The following day, membranes were washed three times (5 minutes per wash) in TBS-T. Membranes were then incubated in secondary antibody (rabbit anti-horse IgG [H&L] DyLight 800 [Rockland Immunochemicals]) at 1 in 15,000 in blocking solution for 2 hours at room temperature on an orbital shaker. Membranes were washed a further three times in TBS-T and once in TBS, prior to imaging for

2 minutes in the 700 and 800 nm channels on an Odyssey Fc Imaging System. All images were obtained using the Image Studio software (Version 5.2, LI-COR Biosciences).

1. WHO Expert Committee on Biological Standardization. Sixty-seventh report (TRS1004). Geneva. World Health Organisation. 2017. Annex 5, Guidelines for the production, control and regulation of snake antivenom immunoglobulins. 197–388
2. Gornall AG, Bardawill CJ, David MM. Determination of serum proteins by means of the biuret reaction. *J Biol Chem*. 1949; **177**: 751–66
3. Lomonte B, Fernández J. Solving the microheterogeneity of *Bothrops asper* myotoxin-II by high-resolution mass spectrometry: Insights into C-terminal region variability in Lys49-phospholipase A2 homologs. *Toxicon*. 2022; **210**: 123–131
