# Supplementary material for: African polyvalent antivenom can maintain pharmacological stability and ability to neutralise murine venom lethality for decades post-expiry: evidence for increasing antivenom shelf life to aid in alleviating chronic shortages
Source: BMJ Glob Health. 2024 Mar 13;9(3):e014813. doi: 10.1136/bmjgh-2023-014813 (PMC10941113; doi:10.1136/bmjgh-2023-014813)
Supplement: Supplementary data [file bmjgh-2023-014813supp001.pdf]

Supplementary Figure 1

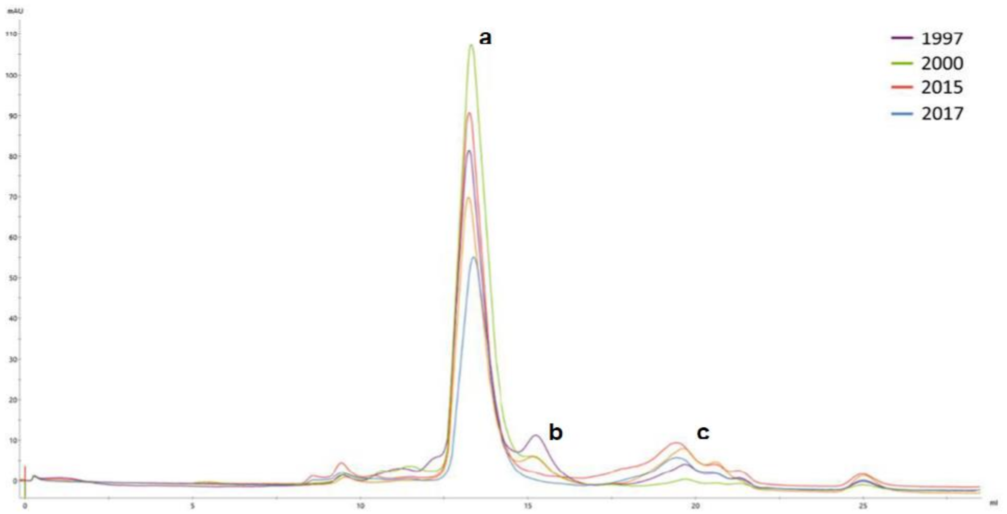

Supplementary Figure 1. Gel Filtration traces of four SAIMR polyvalent batches which expired in 1997, 2000, 2015 and 1017. a and b are calibrated vs. markers: a is 107 kDa, b is 42 kDa. c represents small molecular weight ‘material’, likely peptides of various sizes.
