# Supplementary material for: African polyvalent antivenom can maintain pharmacological stability and ability to neutralise murine venom lethality for decades post-expiry: evidence for increasing antivenom shelf life to aid in alleviating chronic shortages
Source: BMJ Glob Health. 2024 Mar 13;9(3):e014813. doi: 10.1136/bmjgh-2023-014813 (PMC10941113; doi:10.1136/bmjgh-2023-014813)
Supplement: Supplementary data [file bmjgh-2023-014813supp002.pdf]

Supplementary Figure 2

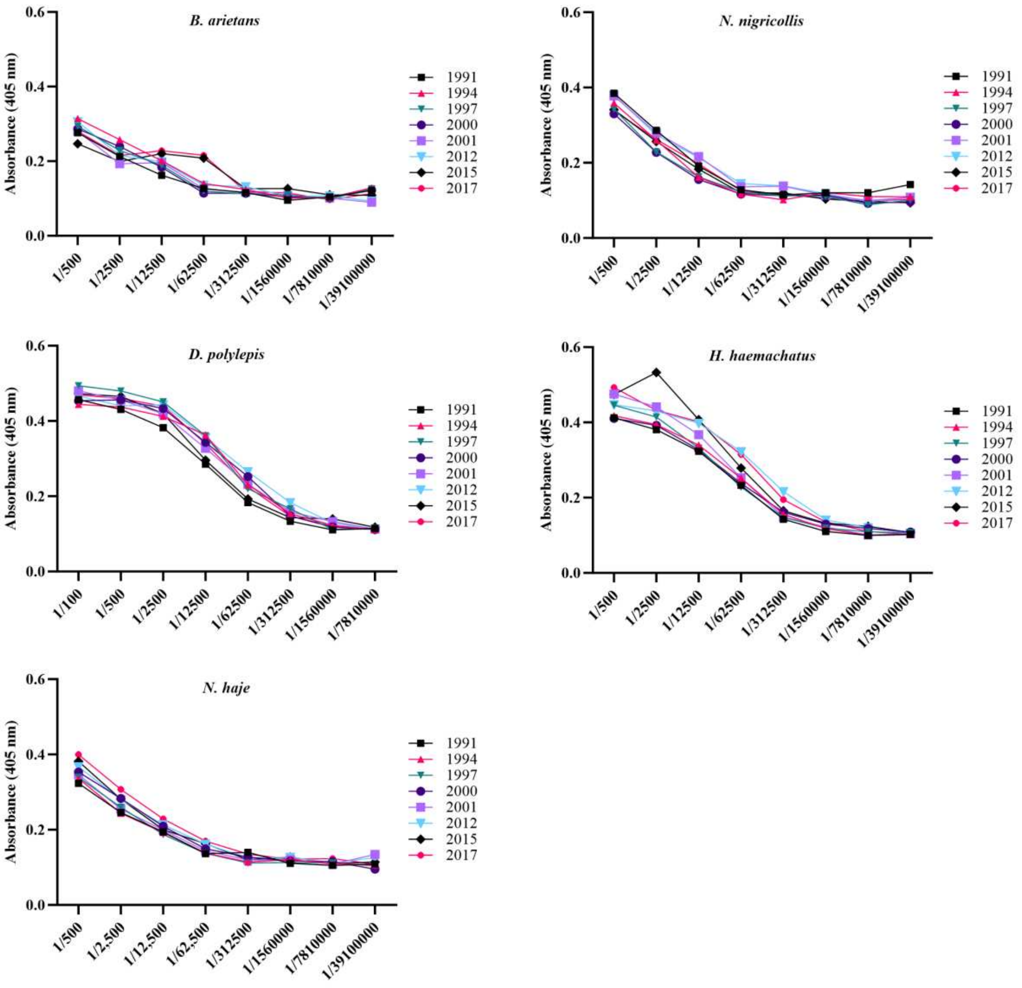

Supplementary Figure 2 The IgG reactivity (titre) of expired SAIMR polyvalent antivenoms against five sub-Saharan Africa venoms determined by titration ELISA. Results are the mean of three replicates with error bars representing standard deviation (SD). Error bars are not shown where SD is smaller than data point. The reactivity of control, naïve horse IgG to each venom was consistently low at each dilution for each venom.
