# Supplementary material for: African polyvalent antivenom can maintain pharmacological stability and ability to neutralise murine venom lethality for decades post-expiry: evidence for increasing antivenom shelf life to aid in alleviating chronic shortages
Source: BMJ Glob Health. 2024 Mar 13;9(3):e014813. doi: 10.1136/bmjgh-2023-014813 (PMC10941113; doi:10.1136/bmjgh-2023-014813)
Supplement: Supplementary data [file bmjgh-2023-014813supp003.pdf]

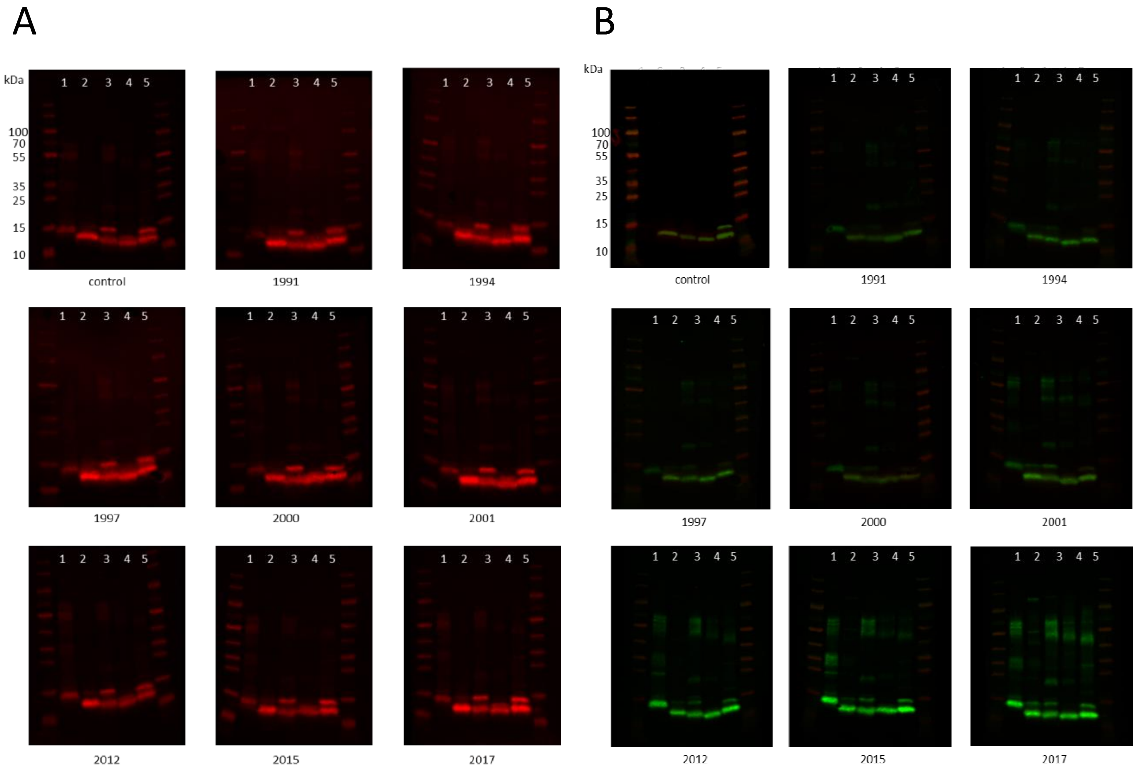

Supplementary Figure S3 Immunboltting of expired antivenoms vs. venoms. A Total protein stains of venoms of blots. **B**. Secondary antibody (rabbit anti-horse IgG [H&L] DyLight 800 [Rockland Immunochemicals]). Blots were scanned at 700nm and 800 nm for 2 minutes in each channel. Unimmunised horse immunoglobulins (BioRad) were used as control. Venoms used are: 1 *B. arietans*, 2 *D. polylepis*, 3 *H. haemachatus*, 4 *N. haje*, and 5 *N. nigricollis.* Blots were stained with TotalProtein Stain 700 (Licor) to show protein loading, and scanned at 700nm for 2 minutes. Years denote which expired antivenom was to be applied to which blot
