# Supplementary material for: African polyvalent antivenom can maintain pharmacological stability and ability to neutralise murine venom lethality for decades post-expiry: evidence for increasing antivenom shelf life to aid in alleviating chronic shortages
Source: BMJ Glob Health. 2024 Mar 13;9(3):e014813. doi: 10.1136/bmjgh-2023-014813 (PMC10941113; doi:10.1136/bmjgh-2023-014813)
Supplement: Supplementary data [file bmjgh-2023-014813supp005.pdf]

Supplementary Table 1

Supplementary Table S1: Ability of expired SAIMR polyvalent antivenoms to neutralise venom imduced lethality in mice[a].

Expiry year	Batch number	Value	*B. arietans*[b]			*N. nigricollis*[c]			*D. polylepis*[c]		
1991	A706 S1	ED_{50} mg/mL	8·5	6·2	11·2	1·0	0·6	1·5	1·4	0·9	3·2
		ED_{50} µL/mg	117.6	161·3	89·3	1000·0	1666·7	666·7	714·3	1111·1	312·5
		ED_{50} µL/mouse	12.9	17·7	9·8	1·1	1·9	0·8	39·4	61·3	17·3
		P (mg/mL)	6.8	5·0	9·0	0·7	0·4	1·0	0·9	0·6	2·1
1994	D04446	ED_{50} mg/mL	14.2	11·0	19·2	0·8	0·6	1·1	0·9	0·6	1·3
		ED_{50} µL/mg	70.4	90·9	52·1	1250·0	1666·7	909·1	1111·1	1666·7	769·2
		ED_{50} µL/mouse	7.7	10·0	5·7	1·4	1·9	1·0	61·3	92·0	42·5
		P (mg/mL)	11.4	8·8	15·4	0·5	0·4	0·7	0·6	0·4	0·9
1997	G03146	ED_{50} mg/mL	9.8	7·4	12·8	1·4	0·6	1·9	1·5	1·1	2·6
		ED_{50} µL/mg	102.0	135·1	78·1	714·3	1666·7	526·3	666·7	909·1	384·6
		ED_{50} µL/mouse	11.2	14·9	8·6	0·8	1·9	0·6	36·8	50·2	21·2
		P (mg/mL)	7.8	5·9	10·2	0·9	0·4	1·3	1·0	0·7	1·7
2000	JO6646	ED_{50} mg/mL	17.7	15·9	21·0	1·1	0·8	1·7	1·4	0·9	3·2
		ED_{50} µL/mg	56.5	62·9	47·6	909·1	1250·0	588·2	714·3	1111·1	312·5
		ED_{50} µL/mouse	6.2	6·9	5·2	1·0	1·4	0·7	39·4	61·3	17·3
		P (mg/mL)	14.2	12·7	16·8	0·7	0·5	1·1	0·9	0·6	2·1
2001	K04846	ED_{50} mg/mL	13.5	9·6	19·6	0·9	0·4	1·6	0·9	0·6	1·3
		ED_{50} µL/mg	74.1	104·2	51·0	1111·1	2500·0	625·0	1111·1	1666·7	769·2
		ED_{50} µL/mouse	8.1	11·5	5·6	1·3	2·9	0·7	61·3	92·0	42·5
		P (mg/mL)	10.8	7·7	15·7	0·6	0·3	1·1	0·6	0·4	0·9
2012	X02646	ED_{50} mg/mL	25.3	22·0	29·9	1·6	0·7	2·3	2·4	2·2	2·7
		ED_{50} µL/mg	39.5	45·5	33·4	625·0	1428·6	434·8	416·7	454·5	370·4
		ED_{50} µL/mouse	4.3	5·0	3·7	0·7	1·6	0·5	23·0	25·1	20·4
		P (mg/mL)	20.2	17·6	23·9	1·1	0·5	1·5	1·6	1·5	1·8
2015	BB01446	ED_{50} mg/mL	23.1	22·0	25·3	1·3	0·7	1·8	1·5	1·1	2·6
		ED_{50} µL/mg	43.3	45·5	39·5	769·2	1428·6	555·6	666·7	909·1	384·6
		ED_{50} µL/mouse	4.8	5·0	4·3	0·9	1·6	0·6	36·8	50·2	21·2
		P (mg/mL)	18.5	17·6	20·2	0·9	0·5	1·2	1·0	0·7	1·7
2017	BF00546	ED_{50} mg/mL	17.9	12·8	22·5	0·9	0·7	1·5	2·4	2·1	2·7
		ED_{50} µL/mg	55.9	78·1	44·4	1111·1	1428·6	666·7	416·7	476·2	370·4
		ED_{50} µL/mouse	6.1	8·6	4·9	1·3	1·6	0·8	23·0	26·3	20·4
		P (mg/mL)	14.3	10·2	18·0	0·6	0·5	1·0	1·6	1·4	1·8

a. Intravenous values (premixed venom/antivenom)
b. Challenge dose = 5 x LD_{50}
c. Challenge dose = 3 x LD_{50}
